# I-131 Radiation-Induced Myelosuppression in Differentiated Thyroid Cancer Therapy

**DOI:** 10.4274/mirt.59454

**Published:** 2018-06-07

**Authors:** Stephan Probst, Gad Abikhzer, Guillaume Chaussé, Michael Tamilia

**Affiliations:** 1 Jewish General Hospital, Clinic of Nuclear Medicine, Montreal, Canada; 2 Jewish General Hospital, Clinic of Endocrinology, Montreal, Canada

**Keywords:** Iodine radioisotopes, bone marrow, thyroid neoplasms

## Abstract

Radioactive iodine (RAI) treatment of differentiated thyroid cancer has been used in clinical practice for almost 60 years and is generally accepted to be a safe and efficacious treatment. Severe toxicity in the form of radiation pneumonitis, sometimes progressing to fibrosis, and bone marrow suppression are reported but remain rare. We present a case of severe myelosuppression requiring hospitalization and transfusion support in an otherwise well, young female patient who had received 175 mCi I-131 for low-volume micronodular lung disease one month prior, with a cumulative lifetime administered activity of 575 mCi. The most important risk factors for myelosuppression following RAI are the activity received, the amount of functioning thyroid tissue present, and the lifetime cumulative activity received.

## Abstract (tr)

Radyoaktif iyot (RAİ) diferansiye tiroid kanseri tedavisinde yaklaşık 60 yıldır klinik uygulamada kullanılmaktadır ve genellikle güvenli ve etkili bir tedavi olarak kabul edilir. Fibrozise ilerleyebilen radyasyon pnömonisi ve kemik iliği süpresyonu gibi ciddi yan etkiler bildirilmiştir ancak nadiren rastlanır. Bu makalede bir ay önce düşük volümlü mikronodüler akciğer hastalığı için 175 mCi I-131 tedavisi, hayat boyu uygulanan kümülatif aktivite 575 mCi, aldıktan sonra ciddi miyelosüpresyon nedeniyle hastane yatışı ve transfüzyon desteği ihtiyacı olan genç bir kadın hasta sunulmaktadır. RAİ sonrası miyelosüpresyon için en önemli risk faktörleri alınan aktivite miktarı, mevcut fonksiyonel tiroid dokusu ve alınan hayat boyu kümülatif aktivite miktarıdır.

## Introduction

Well-differentiated thyroid cancers (DTC), although they generally confer a good prognosis, sometimes follow an aggressive disease course (1,2). When they do occur, DTC metastases are best treated with high-dose I-131 if surgical resection is impossible or otherwise ill-advised (3). Radioiodine remnant ablation and high-dose RAI treatment of metastatic disease has been shown to improve disease-specific mortality in intermediate and high-risk patients (1). Curative-intent treatment can be envisaged with low-volume micronodular lung or small nodal disease although bone metastases are often resistant to treatment (3). The goal of RAI therapy is to deliver very high doses (>100 Gy) to the tumor while keeping the dose to the bone marrow below 2 Gy (4). Myelosuppression is the most important and dose-limiting toxicity of high-dose I-131 therapy but remains rare in clinical practice. Bone marrow suppression is dose-dependent and highly related to the volume of functional thyroid tissue and lifetime total activity received (3,5). Pulmonary fibrosis is another potentially fatal complication of high-dose I-131 when the tumor burden in the lungs is important (2). Other more common acute toxicities include nausea and vomiting, epigastric discomfort and sialadenitis; however, these are self-limiting conditions (2). Generally, the accepted safe tolerance for the blood (or bone marrow) is a total absorbed dose of 2 Gy or, as a surrogate, less than 80 mCi whole-body retention at 48 h with lung metastases and 120 mCi at 48 h without lung lesions (6,7). Dosimetry, although described in the literature, involves estimates and assumptions, remains time-consuming and expensive, and is not routinely performed at our institution (1). However, dosimetric calculations can lead to the safe administration of higher treatment doses for about a quarter of patients; and conversely can identify the small number of patients in which empiric doses must be reduced (4). Most centers, including ours, have gravitated towards an empiric, fixed-dose treatment regimen using 30-200 mCi for RAI. Although certain authors have suggested that empiric dosing often exceeds maximum tolerated activities in older patients, most institutions do not report clinically significant adverse outcomes (2,6). In keeping with our experience, some authors note that while myelosuppression is common in patients treated with single-treatment activities up to 300 mCi, it is almost invariably mild (2).

## Case Report

All of the procedures and treatments described in the case report have been performed according to clinical practice standards at our institution after obtaining the patient’s informed consent. Following the initial diagnosis, the patient underwent a total thyroidectomy and lymph node dissection for a 5 cm x 3.5 cm x 2.2 cm diffuse sclerosing variant of papillary thyroid cancer on Feb 9^th^ 2000, at age 19. There were positive margins and all eleven sampled lymph nodes were positive for malignancy. The patient was thyroglobulin antibody-positive and further thyroglobulin measurements were not performed. She underwent radioiodine remnant ablation with 150 mCi I-131 on April 18th 2000 and follow-up post-therapy whole-body scanning revealed no iodine-avid disease outside of the thyroid bed. Subsequent whole-body I-131 scans and high resolution ultrasound of the neck remained negative and the patient was considered to be disease-free until December 2004 when routine iodine-scintiscanning revealed abnormal foci of uptake in the thyroid bed, the left neck and the superior mediastinum. The patient was admitted for high dose I-131 and received 150 mCi on September 17^th^ 2004. Post-therapy scanning revealed the same three pre-treatment foci, and additional uptake at both lung bases, compatible with functioning DTC lung metastases. In 2005 and 2006 serial chest CTs demonstrated slowly progressing scattered milimetric lung nodules bilaterally. Several ultrasound scans of the neck failed to show any local disease. The patient was retreated for progressive micronodular lung metastases with 100 mCi I-131 on September 6^th^ 2006. Post-therapeutic scanning revealed persistent but improved cervical foci and diffuse increased uptake in both lungs. In view of false-negative tracer-dose I-131 scanning, a decision was taken at this time to re-treat the patient in 9 months’ time empirically with high-dose radioiodine. She then received 175 mCi I-131 on July 24^th^ 2007, for a total lifetime activity of 575 mCi. Post-therapy scanning approximately 1 week later again showed lung uptake, but it was much improved as compared to 2006. No complete blood count (CBC), chemistry panel or other blood work was ordered at the time of admission for RAI treatment as this was not part of the institution’s protocol, and the patient was discharged in good clinical condition to follow up with her endocrinologist. About one month post-treatment on August 26^th^ 2007, the patient presented to her local community hospital complaining of fatigue, heavy menses, epistaxis, headache and multiple pre-syncopal episodes in the preceding week. Upon investigation she was found to have pancytopenia with a platelet count of 12.000 per microliter, a hemoglobin (Hg) of 85 g/L, a white blood cell (WBC) count of 1.5 per microliter and neutrophils of 0.7 per microliter. The first CBC performed at our institution on the following day is explained in detail in Table 1. Although no recent values were available for a trend comparison, a CBC in 2002 (5 years prior) was completely within normal limits and the most recent CBC in 2004 (3 years prior) was remarkable only for a slightly low platelet count of 112.000 per microliter (reference range 150-400.000 per microliter). Her TSH was appropriately suppressed and free T4 was just above the upper limit of normal. Blood pressure (112/71 mmHg), heart rate (64 BPM) and serum biochemistry were normal and she was afebrile when she was transferred to our institution for further treatment. Upon admission, packed red blood cell (RBC) and platelet transfusions were given and the granulocyte colony-stimulating factor analog, filgrastim (300 mcg SQ QD) and erythropoietin (48.000 units SQ QWeek) were started. The Hg and WBC trends from the admission are illustrated in Graphic 1; the jumps in Hg represent RBC transfusions. The full virological work-up was negative. Vitamin B12 and folate levels were normal. The immunoglobulin profile, beta-2 microglobulin and iron studies were unremarkable. The patient had an inappropriately low level of circulating reticulocytes, given her anemia. The hematology consult team, having failed to identify any other cause of the pancytopenia, settled on a working diagnosis of RAI-induced bone marrow suppression. The patient’s admission was complicated by a febrile neutropenic episode which was empirically treated for a number of days with ticarcillin/clavulanate and gentamycin; however, a source of infection was never identified and the fever resolved. A single episode of hypotension also arose and responded well to intravenous fluid challenge. RBCs, platelets and WBCs showed slow recovery towards the end of the admission period. She was discharged 17 days later in good condition with blood counts still below normal, to be followed up closely with her endocrinologist and other specialists. Extensive post-discharge testing was negative, including anti-nuclear antibodies, physical and chemical urinalysis, Westergren sedimentation rate, erythrocyte sedimentation rate, C-reactive protein and extractable nuclear antigen antibody screening. Follow-up consultations with hematology, rheumatology and other specialists failed to turn up any alternative diagnoses such as connective tissue diseases which could explain the cytopenia. The cytopenias spontaneously recovered as assessed in subsequent follow-ups (Graphic 1). The last thyrogen-stimulated 2 mCi I-123 scan in October 2014 did not demonstrate active thyroid tissue. At the last clinical follow-up in June 2015, the patient had no evidence of recurrence of thyroid disease or signs of marrow suppression.

## Discussion

Following oral RAI administration, blood radioiodine concentrations follow 2 distinct phases. In the first phase (0-48 h) inorganic free iodine is absorbed through the GI mucosa and is quickly removed from plasma by thyroid tissue and the kidneys. The second phase (2-10 d) sees a rise in plasma RAI activity as radioiodinated thyroid hormone is released by functioning tissue (5). The area under the curve of the free inorganic iodine time-activity profile in phase 1 is variable and related to the total body iodine stores and renal function. However, due to the relatively brief rise and fall of plasma radio-iodine concentrations in phase 1, the dose to the bone marrow is largely related to the amount of radioiodinated thyroid hormone formed and released in the second phase. The peak of the second phase is also highly variable, relating to the amount of functioning thyroid tissue present, both malignant and benign. Therefore, the burden of tumor (or normal functioning thyroid tissue in the case of a first ablative dose post-thyroidectomy) is the major factor in determining the total dose to the bone marrow for a given administered activity (6). As opposed to exogenous thyroid hormone withdrawal, rhTSH stimulation seems to increase overall RAI clearance rates by as much as 30%, although the clinical implications of this are unclear (6,8,9). Administration of high doses of I-131 as opposed to limited dose fractionation has several purported advantages (1). The general consensus is that the first treatment is potentially the most efficacious as radio-resistant clones are naturally selected by the exposure (2). Tumor heterogeneity is an important consideration, as RAI-concentrating abilities of DTC can vary widely within a single lesion (1). The loss of crossfire effect following the destruction of RAI-avid tissue will tend to diminish the effectiveness of subsequent RAI administrations (1,2). Also, dedifferentiation with loss of radioiodine-avidity and finally anaplastic transformation are poor-prognosis events in DTC (2). The risk of leukemia, aplastic anemia and myelodysplastic syndrome post high-dose RAI seems to be more strongly correlated with cumulative dose to the bone marrow than with the activity of any given treatment. This too would support the notion that fewer doses with more activity per dose is likely to be beneficial to the patient (1,10). However, there have been data suggesting that lifetime cumulative doses of RAI have a protracted and clinically significant impact on peripheral blood counts (5). In our patient, the last dose of radioiodine (175 mCi) and the burden of functioning disease as assessed by post-therapeutic scintiscanning was not out of the ordinary for our institution. Her CBCs in the years prior had been normal or near-normal. This is the first patient we have encountered who presented following RAI therapy with such profound myelosuppression. At our hospital, larger doses are given for larger tumor volumes and to older patients with putatively less bone marrow reserve on a routine basis. It must be noted that at a total lifetime administered activity of 575 mCi, our patient was on the higher-end of the 600-800 mCi lifetime ceiling suggested by some authorities. This total lifetime administered activity is also on the very high end of the range which we encounter at our institution. Extensive work-up failed to identify another cause for our patient’s pancytopenia. Although the high variability of bone marrow absorbed dose in relation to a given activity of I-131 can be influenced by many factors, the profound hematologic toxicity encountered in this patient lends credence to the theory that the cumulative effect of multiple doses can persist over time (5). In conclusion, although there is no way to definitively diagnose the condition, we suspect that our patient was suffering from radiation-induced bone marrow suppression secondary to RAI administration. The most important risk factors for myelosuppression following RAI are the activity received, the amount of functioning thyroid tissue present, and the lifetime cumulative activity received. It may therefore be wise to monitor patients’ blood counts more closely when they: 1) Are receiving more than 150 mCi and have a significant RAI-avid tumor burden as proven by prior scintiscanning or 2) Have a cumulative lifetime dose approaching 500 mCi or more. More research will be needed to fully elucidate these risk factors and the impact on potential RAI-induced myelosuppression.

## Figures and Tables

**Table 1:**
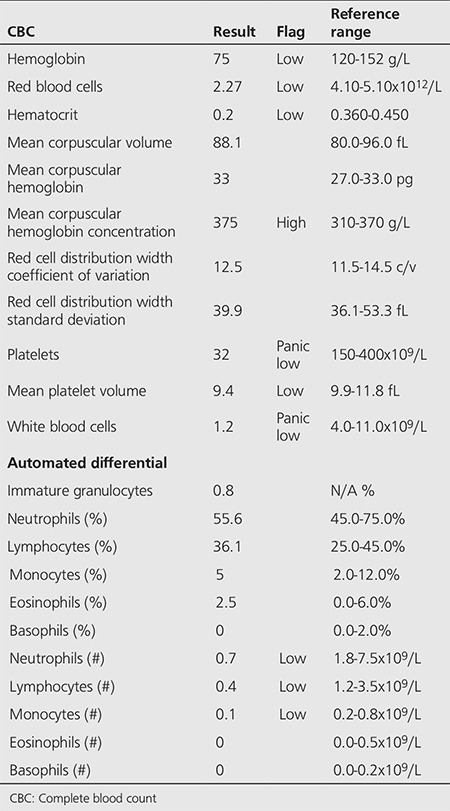
Complete blood count on presentation

**Graphic 1:**
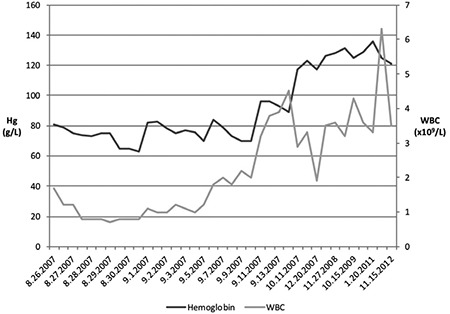
Hematologic parameters *WBC: White blood cells, Hg: Hemoglobin*
